# Interplay between ΔNp63 and miR-138-5p regulates growth, metastasis and stemness of oral squamous cell carcinoma

**DOI:** 10.18632/oncotarget.15752

**Published:** 2017-02-27

**Authors:** Zehang Zhuang, Nan Xie, Jing Hu, Pei Yu, Cheng Wang, Xingxue Hu, Xiaozhe Han, Jinsong Hou, Hongzhang Huang, Xiqiang Liu

**Affiliations:** ^1^ Guangdong Provincial Key Laboratory of Stomatology, Guangzhou, China; ^2^ Department of Oral and Maxillofacial Surgery, Guanghua School and Hospital of Stomatology, Sun Yat-Sen University, Guangzhou, China; ^3^ Department of Oral Pathology, Guanghua School and Hospital of Stomatology, Sun Yat-Sen University, Guangzhou, China; ^4^ Department of Immunology and Infectious Disease, The Forsyth Institute, Cambridge, MA, USA; ^5^ Division of General Practice and Materials Science, The Ohio State University College of Dentistry, Columbus, OH, USA

**Keywords:** oral squamous cell carcinoma, ΔNp63, miR-138-5p, metastasis, stemness

## Abstract

TP63 acts as a master regulator in epithelia development and in the progression of various cancers, but its role in oral cancer pathogenesis remains unknown. This study aimed to explore the role of TP63 in the progression of oral squamous cell carcinoma (OSCC). This study shows that ΔNp63, the predominant isoform of TP63, is significantly upregulated in OSCC tissues and cell lines compared with their normal counterparts, and its expression is closely correlated with pathological differentiation, lymph node metastasis and clinical stage in patients with OSCC. The overexpression of ΔNp63 promotes growth, metastasis and stem-like properties in OSCC cells, and ΔNp63 depletion significantly represses OSCC cellular phenotypes *in vitro* and *in vivo*. The ΔNp63 isoform transcriptionally suppresses miR-138-5p expression; restoration of miR-138-5p expression partially abolishes the effect of upregulating ΔNp63. This study also demonstrates that miR-138-5p directly targets ΔNp63, resulting in crosstalk with ΔNp63. The correlation between ΔNp63 and miR-138-5p was further validated in OSCC tissues and was found to be significantly associated with the prognosis of patients with OSCC. Therefore, our data reveal that the interplay between ΔNp63 and miR-138-5p promotes OSCC progression by regulating cell growth, metastasis and stemness.

## INTRODUCTION

Oral squamous cell carcinoma (OSCC) is a common, morbid, and frequently lethal malignancy. According to the statistics of the American Cancer Society (http://seer.cancer.gov/statfacts/html/oralcav.html), an estimated 48,000 new OSCC cases were diagnosed in 2016, and these cases composed 3% of all new malignancies [1]. Despite advances in knowledge of OSCC pathogenesis, the survival rate has shown little improvement over the past decades [2]. Thus, a deeper understanding of OSCC pathogenesis is needed for the development of effective therapeutic approaches.

TP63, a homolog of the TP53 family of transcription factors, plays a pivotal role in epithelial development. The TP63 gene encodes two main isoforms, TAp63 and ΔNp63. TAp63 contains an N-terminal transactivation (TA) domain, whereas ΔNp63 lacks the TA domain. Additionally, alternative splicing, which occurs at the 3′ end of the RNA transcripts of TAp63 and ΔNp63, produces three different C-termini (α, β and γ); they have distinct biological functions that have not been fully elucidated [3, 4]. ΔNp63, the most abundant TP63 protein product, promotes the self-renewal of basal keratinocytes in stratified squamous epithelia [5], whereas TP63-knockout mice show profound developmental defects in their stratified epithelia [6, 7].

The role of TP63 in cancer biology remains controversial. TAp63 isoforms function as TP53 homologs through their full-length N-terminal TA domains to induce apoptosis and cell cycle arrest [8, 9]. However, ΔNp63 isoforms act as antagonists of the TP53 family members by inhibiting apoptosis and promoting cell proliferation [10, 11]. ΔNp63 is commonly overexpressed in epithelial cancers, including prostate cancer, breast cancer and head and neck cancer (HNC) [11–14]. Recent studies indicate that ΔNp63 is involved in regulating the stemness properties of cancer cells, and is closely associated with tumor initiation and progression [15, 16]. Genome-scale studies confirm that TP63 amplification is one of the most frequent genomic alterations in HNC, which implicates the dysregulation of TP63 as a major driver of HNC carcinogenesis [17]. Clinically, an elevated TP63 level is significantly associated with tumor progression, shorter survival times, and resistance to radiotherapy in OSCC patients[18–20]. Moreover, ΔNp63 often performs oncogenic functions to promote tumor cell proliferation, migration and inflammation *in vitro* and *in vivo* [21]. Together, these data suggest that TP63, and likely ΔNp63, are important regulators of OSCC development and progression. However, the molecular mechanism by which TP63 acts in OSCC pathogenesis remains elusive.

MicroRNAs (miRNAs) are endogenous small non-coding RNAs that post-transcriptionally regulate target gene expression. Studies show that dysregulation of specific miRNAs, including miR-21 [22], miR-138 [23], miR-200b [24] and miR-320a [25], contributes to OSCC growth, invasion, metastasis and chemoresistance. Downregulation of miR-204 in OSCC-derived cancer stem cells has been reported; up-regulation of miR-204 suppresses cancer stemness and epithelial-mesenchymal transition (EMT) properties by targeting SLUG and SOX4 [26]. TP63 regulates a subset of miRNAs in multiple human cancers. ΔNp63 promotes metastatic dissemination by repressing miR-527 and miR-665 [27]. Moreover, ΔNp63 suppresses EMT by inducing miR-205 expression in bladder cancers [28]. These findings indicate that miRNAs are closely associated with the TP63 network, although the interplay between TP63 and the miRNAs involved in regulating tumor progression remains unclear.

The aim of this study was to explore the roles of TP63 and its protein product, ΔNp63, in OSCC progression. Here, we report that TP63 and ΔNp63 regulate tumor growth, metastasis and stemness via miR-138-5p. The loss of miR-138-5p expression promotes oncogenesis in part by targeting ΔNp63. Importantly, the ΔNp63 interaction with miR-138-5p significantly promotes OSCC development and progression. Our results suggest that ΔNp63 and miR-138-5p may provide as new theranostic and prognostic markers for OSCC patients.

## RESULTS

### TP63 is upregulated in OSCCs

**Figure 1:**
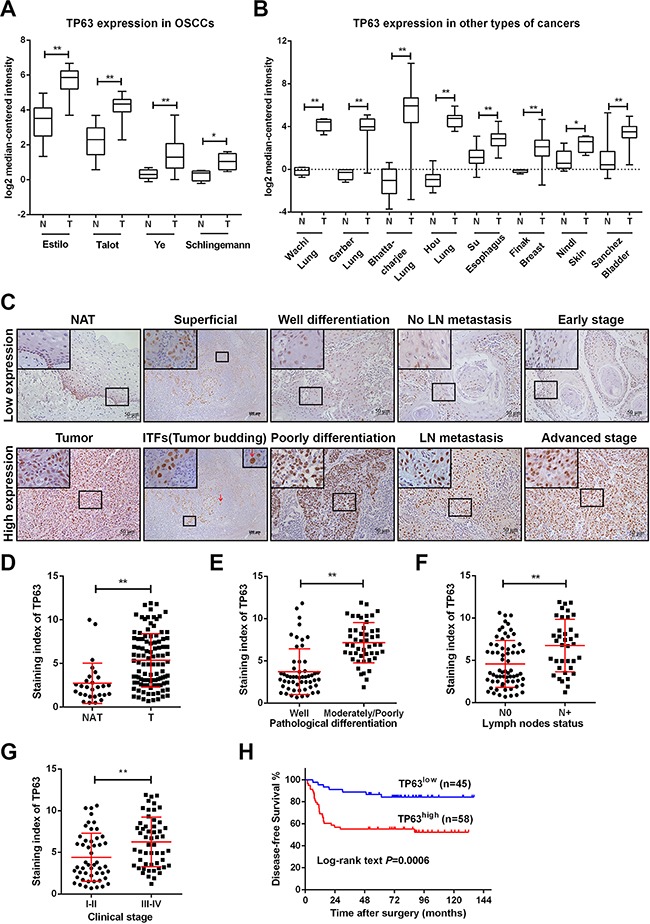
TP63 is upregulated in OSCCs and predicts poor clinical outcomes in OSCC patients **A**. TP63 mRNA levels were significantly upregulated in OSCC per four independent microarrays that were retrieved from Oncomine. **B**. Increased TP63 mRNA expression was revealed in several types of human cancers per Oncomine. Log_2_ median-centered intensity represents the TP63 mRNA expression levels. **C**. TP63 expression in human OSCCs in cohort #1 (n=103) and noncancerous adjacent tissues (NAT, n=28). Representative immunohistochemistry images for TP63 staining in NAT, different localization in one OSCC tissue and OSCC tissues from various pathological differentiation, lymph nodes statuses and clinical stages are shown. Red arrows represent tumor budding cells. The representative images of low expression (upper panel) or high expression (lower panel) of TP63 are shown. Original magnification 400×. **D-G**. A vertical scatter plot is presented to demonstrate the relative expression levels of TP63 in NATs and OSCCs (D), OSCC tissues from patients with different pathological differentiation (E), lymph nodes metastasis statuses (F) and disease stages (G). **H**. Kaplan-Meier curves for the disease-free survival (DFS) of OSCC patients with low TP63 expression (n=45) vs. high TP63 expression (n=58). *P<0.05, **P<0.01.

To investigate the role of TP63 in OSCC pro-gression, we systematically compared TP63 expression levels in OSCCs using the latest microarray datasets in Oncomine (see Methods). The differential expression analysis identified TP63 as a potential candidate that is upregulated in OSCCs (Figure 1A). Moreover, the upregulation of TP63 expression was also demonstrated in various types of solid malignancies, including cancers of the lung, esophagus, breast, skin, and bladder (Figure 1B).

The TP63 has two isoforms, TAp63 and ΔNp63. To validate the Oncomine analysis, we measured TP63, TAp63 and ΔNp63 expression levels in OSCC tissues with different disease severities according to the UICC staging system. OSCC tissues showed higher TP63 levels than did their normal counterparts (noncancerous adjacent tissues, NAT), which was consistent with the Oncomine findings (Figure 1C). A similar ΔNp63 expression pattern was detected in OSCC tumors (Supplementary Figure 1A). However, TAp63 was undetectable in most OSCC tumors, and its level was not significantly different between OSCC tumors and NATs. Notably, the TP63 level was significantly higher in invasive tumor fronts (ITFs) and tumor buddings compared with the superficial or center counterparts (Figure 1C). Furthermore, the relative expression level of TP63 was increased in OSCC cell lines compared with cultured normal oral keratinocytes (NOK). Predictably, ΔNp63 was upregulated in a manner similar to that of TP63 in OSCC cell lines (Supplementary Figure 1B, 1C). We reasoned that ΔNp63 was the predominant TP63 isoform and that TP63 played a role in OSCC progression.

### TP63 is a marker for OSCC progression and prognosis

The average TP63 expression level was significantly increased in OSCC specimens (*P* < 0.001) (Figure 1C, 1D), with a 2-fold increase in the OSCC tissues compared with NAT. To investigate the clinicopathological significance of TP63 expression in patients with OSCC, the median relative expression level of TP63 in the 103 OSCC samples was recommended as the cutoff point for dividing the TP63 levels into a low-expression group and a high-expression group. Correlation analysis showed that TP63 expression closely correlated with pathological differentiation (*P* < 0.001), lymph node (LN) metastasis (*P* =0.001) and clinical stage (*P*=0.004) (Figure 1C, 1E-1G, Table 1). Cervical lymph node metastases developed in 18% of the patients in the TP63 low-expression group but in 50% of the patients in the TP63 high-expression group (Table 1).

**Table 1:** Correlation of TP63 expression with clinical and pathological variables of OSCC patients in cohort #1 (n=103)

Characteristic	Subcharacteristic	n	TP63 expression		
			Low	High	*P*
**Gender**	Male	49	20	29	0.575
	Female	54	25	29	
**Age**	≤55	51	22	29	0.911
	>55	52	23	29	
**Tumor size**	≤4cm	81	33	48	0.247
	>4cm	22	12	10	
**LN metastasis**	Negative	66	37	29	**0.001**
	Positive	37	8	29	
**Pathological differentiation**	Well	54	40	14	**<0.001**
	Moderately/Poorly	49	5	44	
**T classification**	T_1_-T_2_	76	32	44	0.587
	T_3_-T_4_	27	13	14	
**Clinical stage**	I-II	50	29	21	**0.004**
	III-IV	53	16	37	

Abbreviations: LN, Lymph node.

To explore the potential of TP63 as a prognostic marker, we performed a clinical study on 103 OSCC patients. The patients were divided into two groups—“TP63^high^” and “TP63^low^”—based on the TP63 expression levels measurements in their surgically removed primary tumors. Follow-up analyses revealed that high TP63 expression was associated with relapses and metastases in these disease-free patients (log-rank test, *P* = 0.0006). The TP63^high^ group had a significantly higher recurrence rate than the TP63^low^ group per the disease-free survival (DFS) curves from a Kaplan-Meier analysis (Figure 1H). A univariate analysis revealed that pathological differentiation (*P*=0.044), LN metastasis (*P*=0.020), clinical stage (*P*=0.017) and TP63 (*P*=0.002) were significant prognostic factors for patients with OSCC. Furthermore, the prognostic value of TP63 for DFS was significant (p = 0.026) in a multivariable Cox proportional hazards model that was adjusted for pathological differentiation, LN metastasis and clinical stage (Table 2). We reasoned that TP63 expression positively correlated with the metastatic potential of OSCCs and that TP63 may promote OSCC growth and metastasis.

**Table 2:** Cox regression models of patients with OSCC for clinical and pathological parameters (cohort#1, n=103)

Characteristic	Subcharacteristic	Univariate Analysis			Multivariate Analysis		
		HR	95% CI	*P*	HR	95% CI	*P*
**Gender**	Female	1					
	Male	1.293	0.659-2.537	0.454			
**Age**	≤55	1					
	>55	1.042	0.531-2.044	0.905			
**Tumor size**	≤4cm	1					
	>4cm	1.166	0.528-2.576	0.704			
**T classification**	T_1_-T_2_	1					
	T_3_-T_4_	1.427	0.695-2.928	0.333			
**Pathological differentiation**	Well	1			1		
	Moderately/Poorly	2.038	1.019-4.073	**0.044**	1.004	0.437-2.308	0.992
**LN metastasis**	Negative	1			1		
	Positive	2.221	1.132-4.357	**0.020**	1.042	0.391-2.775	0.935
**Clinical stage**	I-II	1			1		
	III-IV	2.396	1.167-4.921	**0.017**	1.729	0.635-4.709	0.284
**TP63 expression**	Low	1			1		
	High	3.854	1.676-8.866	**0.002**	3.253	1.152-9.182	**0.026**

Abbreviations: LN, Lymph node; HR, hazard ratio; 95% CI, 95% confidence interval.

### ΔNp63 promotes OSCC growth and metastasis

**Figure 2:**
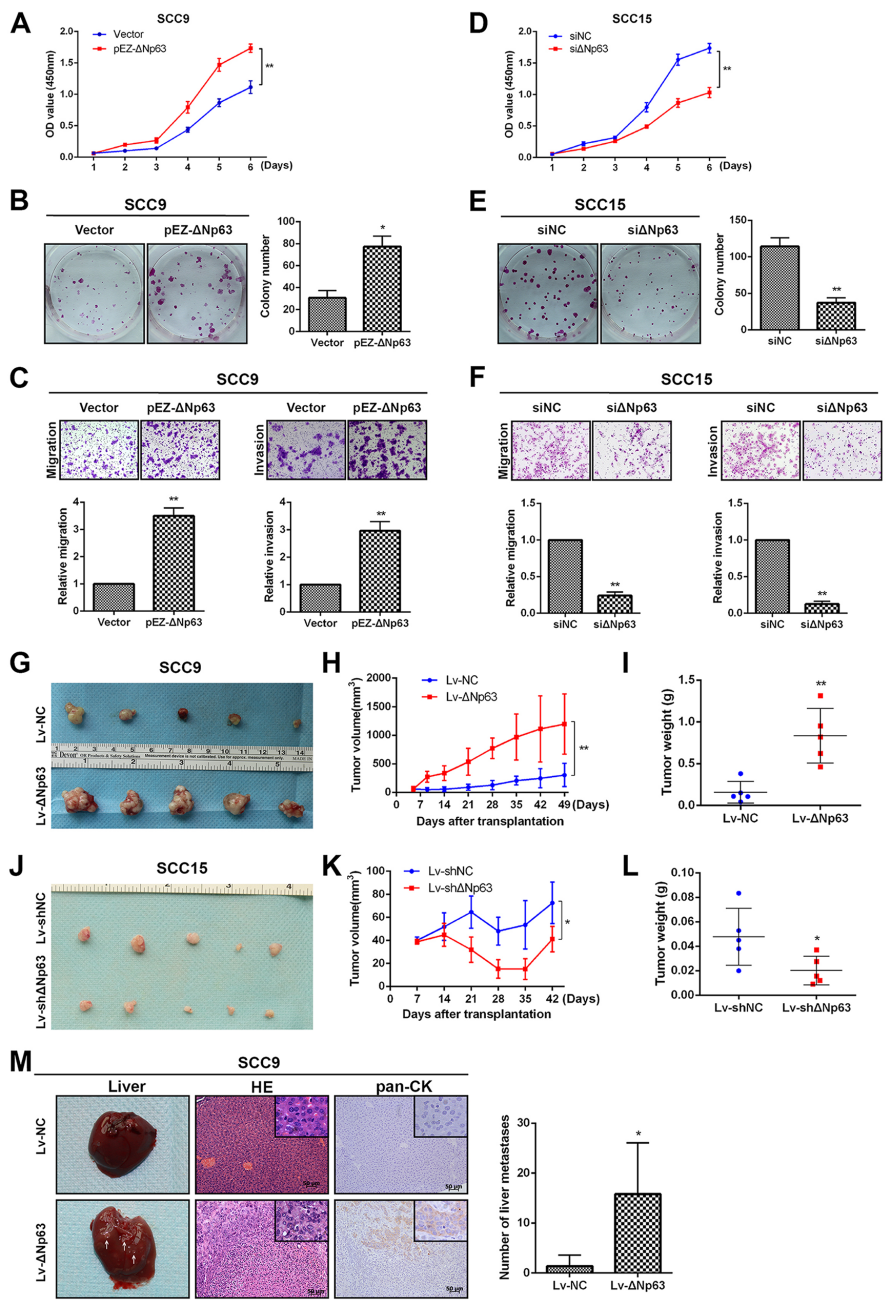
ΔNp63 promotes OSCC growth and metastasis *in vitro* and *in vivo* The impact of ΔNp63 on cell proliferation was determined using CCK-8 **A, D**. and colon-formation assays **B, E**. **C, F**. Transwell assays were used to evaluate the effect of ΔNp63 on cell migration or invasion (upper panel). The relative numbers of cells that migrated or invaded are shown (lower panel). The results were obtained in three independent experiments and are shown as the mean±SD. **G, J**. The effects of ΔNp63 on the tumor xenografts that were generated with subcutaneous injections of the SCC9 or SCC15 stable cells are shown. **H, K**. Growth curves of the tumor volumes, which were measured every 7 days post-injection, are shown. **I, L**. Tumor weights are represented. **M**. Representative photographs of the hepatic metastases, and hematoxylin and eosin (H&E) stains, the immune staining of human pan-CK in metastatic livers of mice (n=5 per group) in the models generated by way of tail vein injection of SCC9 stable cells (left panel). Original magnification 400×. Quantification of the number of liver metastases (right panel). *P<0.05, **P<0.01.

To evaluate the effect of ΔNp63 on SCC9 cells with low endogenous ΔNp63 expression levels, we ectopically expressed ΔNp63 in SCC9 cells. The transfection efficiency in ectopic ΔNp63-expressing cells was evaluated (Supplementary Figure 2A). The upregulation of ΔNp63 increased cell proliferation relative to the controls, as measured by CCK-8 cell proliferation assays (*P*<0.01, Figure 2A) and plate colony-formation assays (*P*<0.05, Figure 2B). Transwell migration and Matrigel invasion assays showed that ectopic expression of ΔNp63 significantly increased SCC9 migration and invasion (*P*<0.01, Figure 2C). However, ectopically expressed TAp63 had no effect on migration or invasion (Supplementary Figure 2B). To investigate whether ΔNp63 silencing impeded OSCC progression, we knocked down ΔNp63 in SCC15 cells, where endogenous expression of ΔNp63 was high (Supplementary Figure 2A). ΔNp63 depletion inhibited cell growth (*P*<0.01, Figure 2D, 2E). ΔNp63 silencing dramatically decreased cell migration and invasion (*P*<0.01, Figure 2F). As expected, the TP63 knockdown had a similarly inhibitory effect on migration and invasion (Supplementary Figure 2C).

To investigate the potential role of ΔNp63 on OSCC tumorigenesis *in vivo*, a nude mouse xenograft model was constructed via subcutaneous injections of SCC9 cells that had been stably infected with Lv-ΔNp63 or Lv-control vectors (Lv-NC). The tumor growth rate in the Lv-ΔNp63 group exceeded that of the control group in the SCC9 transplanted mice (Figure 2G). By the seventh week after the inoculations, the tumor volume and weight in the Lv-ΔNp63 group were significantly higher than those in the Lv-control group (*P*<0.01, Figure 2H, 2I). Additionally, SCC15 cells were infected with lentiviral vectors to establish two stable cell lines that expressed either shRNA against ΔNp63 (Lv-shΔNp63) or a control vector (Lv-shNC). Equal numbers of SCC15 cells that were infected with Lv-shΔNp63 and Lv-shNC were subcutaneously injected (Figure 2J). The tumor volume and weight in the Lv-shΔNp63 group were significantly lower than those in the Lv-shNC group (*P*<0.05, Figure 2K, 2L).

Hepatic metastasis was also assessed in the xenograft tumor models that were generated through tail vein injections of SCC9 cells that had been infected with Lv-ΔNp63 or Lv-NC. As shown in Figure 2M, both the H&E staining and immune staining of pan CK validated that the hepatic metastases were derived from the tail vein injected tumor cells. The number of mice with liver metastases in the Lv-ΔNp63 group (5/5) exceeded that of the mice in the Lv-NC group (2/5). Moreover, our assessment of the number of metastatic nodules in each liver revealed that the number of hepatic metastatic nodules (12, 14, 4, 17, and 32) in the mice from the Lv-ΔNp63 group was clearly higher than that in the mice of the Lv-NC group (2 and 5 metastatic nodules) (P =0.0153).

Collectively, the *in vitro* migration and invasion assays and the *in vivo* tumorigenesis and metastasis assays, which utilized ectopic expression of ΔNp63 in SCC9 cells and silencing of endogenous ΔNp63 in SCC15 cells, indicate that ΔNp63 promotes OSCC growth and metastasis.

### ΔNp63 promotes stem-like cell properties

**Figure 3:**
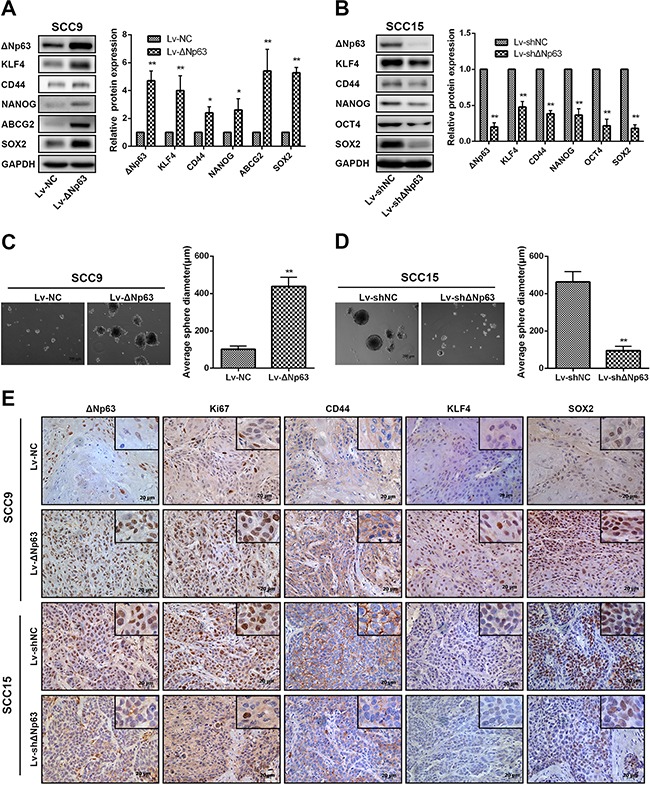
ΔNp63 promotes stem-like cell properties in OSCC cells **A, B**. The impact of ΔNp63 on the expression of representative stem cell markers was assessed by Western blot (left panel). Quantification of the relative protein expression levels is shown (right panel). **C, D**. Representative images of the tumor spheres formed by SCC9 or SCC15 stable cells lines after culturing for 2 weeks (left panel). Statistical analysis of the average diameters of the spheres (right panel). Original magnification 50×. **E**. Expression of the ΔNp63, Ki67, CD44, KLF4, and SOX2 proteins in the transplanted tumors was detected by IHC, and representative micrographs are presented. Original magnification 400×.

Stem-like cell properties are important factors for cancer progression and metastasis, and TP63 has been implicated in the stemness properties of stratified epithelia and cancers. We reasoned that ΔNp63 participated in OSCC stem-like cell phenotypes. We first examined the impact of ΔNp63 on the expression of representative stem cell markers using the aforementioned ΔNp63 depletion or overexpression cell models. Compared with the negative control, ΔNp63 overexpression in SCC9-ΔNp63 cells clearly enhanced the levels of KLF4, CD44, NANOG, ABCG2, and SOX2 (Figure 3A). Conversely, SCC15-shΔNp63 cells exhibited significantly decreased levels of KLF4, CD44, NANOG, OCT4, and SOX2 relative to the negative control (Figure 3B). To further study the importance of ΔNp63 in OSCC stem cell activity, we used a sphere-formation assay to evaluate cellular sphere formation in non-adherent serum-free conditions. Compared with the SCC9 control cells, SCC9-ΔNp63 cells showed a marked increase in sphere size (*P*=0.003, Figure 3C). In turn, SCC15-shΔNp63 cells revealed a decrease in the sphere size compared with the SCC15 control cells (*P*=0.004, Figure 3D).

We next investigated the impact of ΔNp63 on the expression of representative stem cell markers using the aforementioned ΔNp63 overexpressed or depletion xenograft models (Figure 2G, 2J). In agreement with the findings of the *in vitro* experiments, SCC9-ΔNp63 xenografts showed significantly increased levels of CD44, KLF4 and SOX2 compared with the SCC9-NC xenografts, while SCC15-shΔNp63 xenograft tumor cells showed decreased levels of CD44, KLF4 and SOX2 compared with the SCC15-NC xenografts (Figure 3E). Furthermore, Ki67 expression was increased in the SCC9-ΔNp63 xenografts and decreased in the SCC15-shΔNp63 xenografts when compared with their negative control group, respectively. Together, these data indicate that ΔNp63 may endow OSCC cells with stem-like cell properties.

### ΔNp63 regulates miR-138-5p

**Figure 4:**
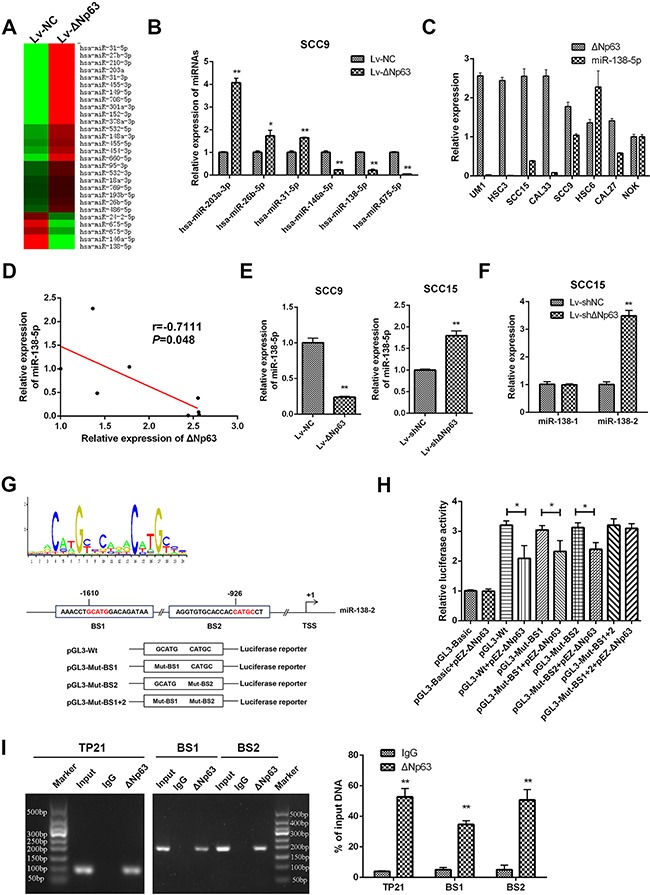
ΔNp63 directly suppresses miR-138-5p expression **A**. The differential miRNA profiles of the SCC9-LvNC and SCC9-LvΔNp63 cells were characterized with a PCR-based miRNA microarray. **B**. Six differentially expressed miRNAs were selected from the miRNA profiles and validated by qRT-PCR. **C**. qRT-PCR analysis showing the miR-138-5p and ΔNp63 expression levels in a panel of 7 oral cancer cells and NOK. **D**. Relationship between ΔNp63 expression and miR-138-5p expression levels in a panel of 7 oral cancer cells and NOK was analyzed by Spearman order correlation. **E**. qRT-PCR analysis showing the miR-138-5p expression levels in the SCC9 or SCC15 stable cell lines. **F**. qRT-PCR analysis showing pre-miR-138 (miR-138-1 and miR-138-2) expression levels in SCC15-shΔNp63 compared with the control group (SCC15-shNC). **G**. TP63 binding motif sequence (upper panel). The schematic diagram represents two putative ΔNp63 binding sites, which occupy the upstream region of the miR-138-2 promoter, and the pGL3-basic plasmid, which contains the wild type (pGL3-Wt) or the mutated binding sites (pGL3-Mut-BS1, pGL3-Mut-BS2, pGL3-Mut-BS1+2) (lower panel). **H**. Luciferase reporter assays were performed in SCC9 cells after co-transfection with pGL3-basic reporters that contained the wild-type or the mutated binding sites and the ΔNp63 plasmid (pEZ-ΔNp63). The data were normalized to data from a simultaneously delivered Renillar luciferase expression plasmid and determined relative to an empty vector (pGL3-basic). **I**. ChIP assays using antibodies against ΔNp63 or the IgG control were performed in SCC15 cells. Binding of ΔNp63 to the TP21 promoter (positive control) and the two binding sites (BS1 and BS2) on miR-138-2 promoter region was confirmed by PCR using specific primers to the binding sites. The PCR gel shows the amplification of the ΔNp63 binding sites (left panel). The qPCR for ΔNp63 binding sites (right panel). All results are presented as the mean±SD obtained from three independent experiments. *P<0.05, **P<0.01.

TP63 has been identified as a transcriptional regulator of miRNAs [29–32]. To explore whether ΔNp63 modulates miRNA expression in OSCCs, we performed a PCR-based miRNA microarray comparison of the stable SCC9-ΔNp63 and SCC9-NC cells. A total of 28 miRNAs (23 upregulated and 5 downregulated) were differentially expressed (DE) between the SCC9-ΔNp63 cells and the SCC9-NC cells (fold change=1.6, Figure 4A). The changes in the expression of several DE-miRNAs, including miR-203a-3p, miR-26b-5p, miR-31-5p, miR-146a-5p, miR138-5p, and miR-675-5p, were validated by qRT-PCR (Figure 4B).

Because miR-138-5p is a frequently downregulated miRNAs, we focused on the possible crosstalk between ΔNp63 and miR-138-5p. We performed qPCR to detect the endogenous expression level of miR-138-5p and ΔNp63 in a panel of 7 oral cancer cell lines and NOK. As shown in Figure 4C and 4D, a negative correlation between the basic expression level of miR-138-5p and ΔNp63 was observed in these cell lines (r=-0.7111, *P*=0.048). Additionally, miR-138-5p was significantly downregulated by ΔNp63 in SCC9 cells, whereas ΔNp63 depletion enhanced miR-138-5p expression in SCC15 cells (*P*<0.01, Figure 4E). We further investigated the expression of the pre-miR-138 (miR-138-1 and miR-138-2) in SCC15-shΔNp63 cells. Knockdown of ΔNp63 increased miR-138-2 expression but had little effect on miR-138-1 expression (Figure 4F). These data suggest that ΔNp63 is an upstream regulator of miR-138-2. To further investigate the potential relationship between miR-138-2 and ΔNp63, we turned to bioinformatics. While analyzing a 2-kb region that was upstream of the transcription start site (TSS) of miR-138-2 using JASPAR (http://jaspardev.genereg.net/), we noted that there were two potential ΔNp63 transcription factor-binding sites (TFBS) that were located within the miR-138-2 promoter (Figure 4G). For convenience, the two TFBSs were named BS1 and BS2. As shown in Supplementary Figure 3, the activity of the miR-138-2 promoter was validated in 293T cells. A reduction in the wild-type miR-138-2 promoter luciferase activity was then observed upon the upregulation of ΔNp63 in SCC9 cells (*P*<0.05, Figure 4H). However, ΔNp63 overexpression did not result in a further reduction in the luciferase activity when BS1 and BS2 were mutated together (Figure 4H). Notably, the luciferase activity remained reduced when ΔNp63 was upregulated even when either of the two binding sites was separately mutated (*P*<0.05, Figure 4H). These data indicate that ΔNp63 binds to a specific promoter TFBS in miR-138-2 and inhibits transcription. Chromatin immunoprecipitation (ChIP) analyses confirmed that ΔNp63 proteins were recruited to both TFBSs within the miR-138-2 promoter (Figure 4I). Therefore, ΔNp63 directly binds to these two TFBSs to attenuate miR-138-2 expression, which leads to reduced miR-138-5p expression.

### ΔNp63 promotes tumor growth, metastasis, and stemness by downregulating miR-138-5p

**Figure 5:**
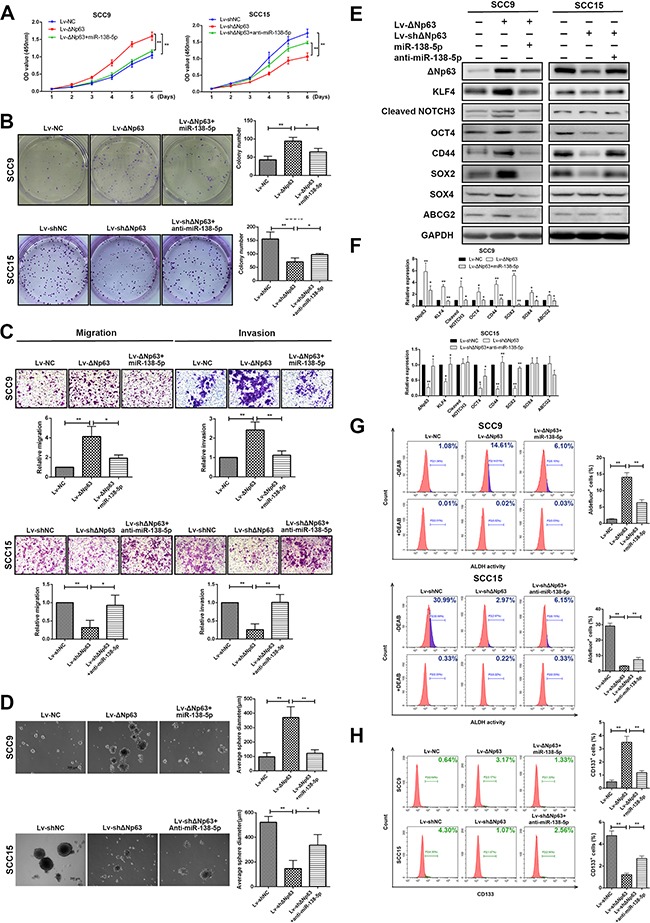
ΔNp63 promotes tumor growth, metastasis, and stemness by downregulating miR-138-5p **A, B**. The effects of ΔNp63 and ΔNp63/miR-138-5p on cell proliferation were determined in SCC9 or SCC15 cells using the CCK-8 (A) and colony formation (B) assays. **C**. The effects of ΔNp63 and ΔNp63/miR-138-5p on the cell migration or invasion of SCC9 or SCC15 cells were measured with transwell assays. **D**. The impact of ΔNp63 and ΔNp63/miR-138-5p on tumor sphere formation and **E**. the exppression of representative stem cell markers. **F**. Protein expression level quantification. **G, H**. Analysis of cancer stem-like cells marker ALDH (G) and CD133 (H) in SCC9 and SCC15 cells. Percentage of ALDH positive cells were calculated against DEAB control. Original magnification 50×. The results are presented as the mean±SD of three independent experiments. *P<0.05, **P<0.01.

To investigate the impact of ΔNp63, as well as its suppression of miR-138-5p expression on growth, metastasis and stemness, overexpression and knockdown strategies were used to modulate miR-138-5p expression levels in SCC9-ΔNp63 and SCC15-shΔNp63 cells. Overexpressing ΔNp63 increased the propensity for proliferation, colony formation, migration and invasion (*P*<0.01, Figure 5A-5C). However, reducing ΔNp63 in SCC15 cells yielded the opposite effect (*P*<0.01, Figure 5A-5C). Importantly, the growth, colony-forming and invasive abilities of ΔNp63 overexpressing SCC9 cells were reduced when miR-138-5p was co-overexpressed. Conversely, the inhibitory effects of ΔNp63 silencing on colony-forming/invasive abilities of SCC15 were also abrogated when anti-miR-138-5p was co-transfected (Figure 5A–5C).

We also investigated whether ΔNp63 and miR-138-5p had a connection with the maintenance of stemness in OSCC cells. Consistent with our previous findings, ΔNp63 overexpression resulted in the acquisition of stemness, which was characterized by increased stem cell sphere-forming capacity (*P*<0.01, Figure 5D) and the increased expression of stem cell markers, such as KLF4, CD44, SOX2, SOX4, and ABCG2 (Figure 5E–5F). Previous studies showed that NOTCH signaling pathway plays a fundamental role in the maintenance of cancer cell stemness in head and neck squamous cell carcinomas (HNSCC) [33, 34]. To evaluate the role of ΔNp63 in the NOTCH pathway activation, we also detected the activated (cleaved form) NOTCH and found that ΔNp63 overexpression increased the expression level of cleaved NOTCH3 (Figure 5E–5F). Additionally, by rescuing miR-138-5p expression, the ΔNp63-induced stemness was significantly abolished (*P*<0.05, Figure 5E-5F). To confirm the significance of ΔNp63 and miR-138-5p in regulating the cancer stemness, we carried out flow cytometry analysis to detect the molecular markers ALDH1 and CD133. As illustrated in Figure 5G-H, ΔNp63-overexpressing SCC9 cells showed a higher number of positive cells, with 14.681% for ALDH and 3.17% for CD133, respectively. However, the positive cells for ALDH and CD133 reduced to 6.10% and 1.33%, respectively, when miR-138-5p expression restored. Similar results were also obtained in SCC15 cells, in which the reduction of stem-like properties through the ΔNp63 knockdown was partially reversed by miR-138-5p downregulation (Figure 5D–5H). These results collectively indicate that ΔNp63 upregulation profoundly enhances tumor growth, metastasis, and stemness, and this enhancement is partially reversed by miR-138-5p.

### miR-138-5p suppresses cancer stemness by directly targeting ΔNp63

**Figure 6:**
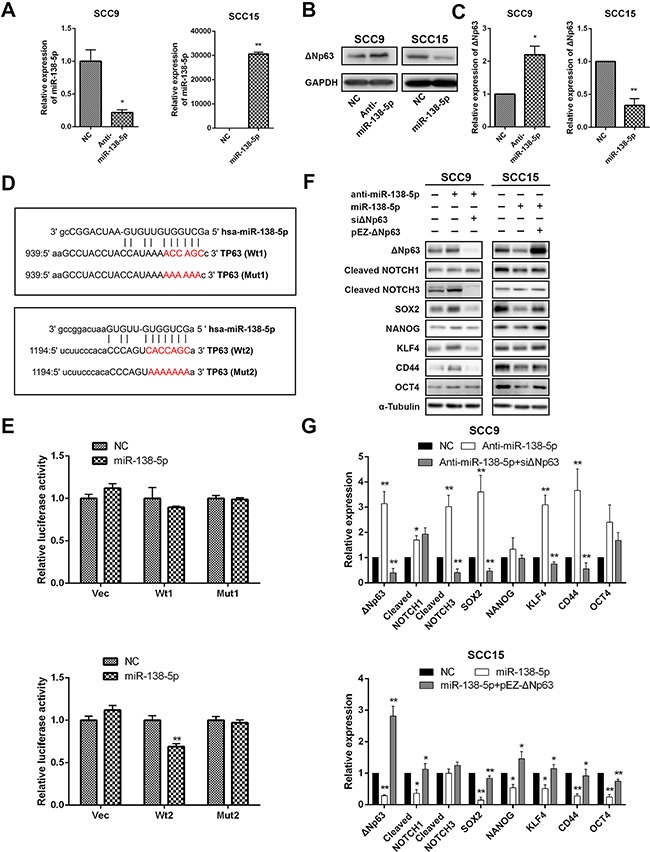
ΔNp63 is a direct target of miR-138-5p **A**. The qRT-PCR shows the miR-138-5p expression levels in cells that were transfected with miR-138-5p mimics or inhibitors as indicated. The miR-138-5p abundance was normalized to U6 RNA. **B**. The Western blot shows the ΔNp63 protein expression level. **C**. Quantification of the ΔNp63 protein expression level. **D**. Schematic diagram of two targeting sites for miR-138-5p in the TP63 3′-UTR per the miRanda database (http://www.microrna.org/). Luciferase reporter plasmids (Vec) with the wild-type (Wt1 and Wt2) or mutant (Mut1 and Mut2) putative target sites were constructed. **E**. The relative luciferase activity that was linked to the TP63 3′-UTR was measured after the transfection of SCC15 cells with miR-138-5p mimics. The data were normalized to Renillar luciferase. **F**. The impacts of miR-138-5p and miR-138-5p/ΔNp63 on the protein expression levels of representative stem cell markers in the indicated cells. **G**. Quantification of the protein expression levels. The results are presented as the mean±SD of three independent experiments. *P<0.05, **P<0.01.

Recent studies have shown that TP63 expression is also regulated by miRNAs in specific cancers, which suppress the proliferation and migration of these cancer cells [35, 36]. To investigate whether miR-138-5p negatively regulates ΔNp63 expression, we compared the ΔNp63 protein levels between miR-138-5p-overexpressing and miR-138-5p-depleted OSCC cells (Figure 6A). Ectopic miR-138-5p expression suppressed the ΔNp63 protein levels, while anti-miR-138-5p activated ΔNp63 expression (Figure 6B, 6C). A bioinformatics analysis identified two candidate miR-138-5p binding sites in the 3′UTR of the TP63 gene (Figure 6D). To determine whether these sites were direct targets of miR-138-5p, the 3′UTR of the TP63 gene, including the two predicted binding sites was subcloned downstream of the firefly luciferase. The luciferase reporters were then co-transfected with miR-138-5p mimics or the negative control (NC) into SCC9 cells. In contrast to the control group, in the cells with ectopic miR-138-5p expression, the luciferase activity of the reporter plasmid that was constructed with the second binding site (Wt2) was reduced (*P*<0.01), whereas mutation (Mut2) of the putative miR-138 seed region in the TP63 3′UTR abrogated the suppression of luciferase activity (Figure 6E). However, miR-138-5p overexpression had no significant effect on the luciferase activity of the reporter plasmid that was generated with the first binding site (Wt1), suggesting that miR-138-5p targeted the second binding site rather than the first. Therefore, miR-138-5p directly targeted TP63 through the identified binding sites in the 3′UTR.

To investigate whether miR-138-5p knockdown promoted the same stem-like phenotypes as ΔNp63, we performed Western blot analyses of the SCC9 cells whose endogenous miR-138-5p expression was suppressed by anti-miR-138-5p. Consistent with ectopic ΔNp63 expression, knockdown of miR-138-5p significantly increased the SOX2, KLF4, NOTCH3 and CD44 protein levels. Conversely, ectopically expressed miR-138-5p in the SCC15 cells decreased the SOX2, NOTCH1, NANOG, KLF4 and CD44 protein levels (Figure 6F, 6G). Interestingly, the stem cell markers reduced by transfection of miR-138-5p can be partially reversed by ΔNp63 restoration (Figure 6F, 6G). Together, our data indicate that miR-138-5p suppresses stem-like cell properties through ΔNp63, which binds to the TP63 binding sites in the miR-138-5p promoter to attenuate miR-138-5p expression.

We observed an inverse change in ΔNp63 mRNA and protein expression when the miR-138-5p level was altered (Supplementary Figure 4A, 4B; Figure 6B, 6C). The partial rescue of ΔNp63 expression was evident with the miR-138-5p/ΔNp63 co-transfection (Figure 6F, 6G). These collective data suggest that miR-138-5p regulates ΔNp63 expression through mRNA degradation and translational silencing, and OSCC progression may be regulated by the interaction between ΔNp63 and miR-138-5p. Our studies suggest that ΔNp63 and miR-138-5p interact with each other, wherein ΔNp63 transcriptionally suppresses miR-138-5p (Figure 4), while miR-138-5p abrogates ΔNp63 expression by directly targeting it.

### A negative correlation between ΔNp63 and miR-138-5p in primary OSCC tissues

**Figure 7:**
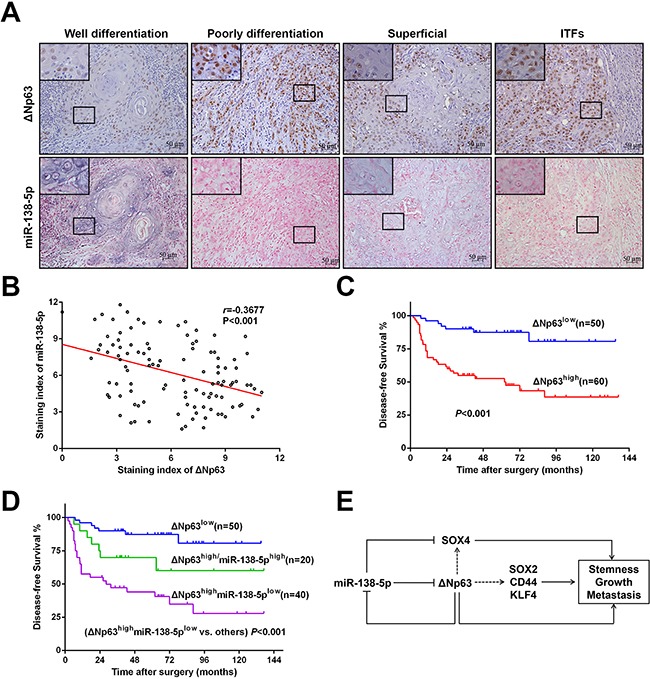
A negative correlation between ΔNp63 and miR-138-5p in primary OSCC tissues **A**. Representative micrographs showing the expression of ΔNp63 and miR-138-5p in OSCC tissues with different pathological differentiation or different localizations from one OSCC tissue specimen. Original magnification 400×. **B**. The relationship between the ΔNp63 expression and miR-138-5p expression levels was analyzed using the Spearman order correlation. **C**. Kaplan-Meier survival curves for the DFS of OSCC patients with low TP63 expression (n=50) vs. high TP63 expression (n=60) **D**. Kaplan-Meier survival curves for the DFS of OSCC patients with low ΔNp63 expression (n=50), high ΔNp63 expression and high miR-138-5p expression (n=20), or high ΔNp63 expression but low-miR-138-5p expression (n=40). The survival differences were analyzed using a log-rank test. **E**. A schematic illustration of the interplay between ΔNp63 and miR-138-5p in regulating OSCC tumorigenesis and metastasis.

To further investigate the *in vivo* correlation of ΔNp63 and miR-138-5p, we compared their expression levels in another independent cohort (#2). The clinicopathological features of the OSCC patients in cohort #2 are described in Supplementary Table 1. As shown in Figure 7A, a relatively low ΔNp63 but high miR-138-5p expression level was frequently observed in well-differentiated OSCC primary tumors. Conversely, high ΔNp63 but low miR-138-5p was observed in poorly differentiated tumors. Compared with the superficial and center parts of the tumors, in the invasive tumor fronts, ΔNp63 expression was higher, and miR-138-5p expression was lower. A negative correlation between ΔNp63 and miR-138-5p was evident (r=-0.3677, *P*<0.001, Figure 7B). Further Kaplan-Meier analysis showed that the ΔNp63^high^ groups had a significantly poorer DFS than the ΔNp63^low^ group (*P*<0.001, Figure 7C), and that the ΔNp63^high^/miR-138^low^ group had the poorest DFS (*P*<0.001, Figure 7D).

## DISCUSSION

In this study, we performed differential analyses of the OSCC samples and noncancerous adjacent tissues using Oncomine. TP63 was identifed as a candidate gene and was confirmed as such using additional datasets from human malignancies, including cancers of the lung, esophagus, breast, skin, and bladder. A study of 103 OSCC patients revealed that TP63 expression level was closely associated with tumor progression. This association was mechanistically explained by the interplay between ΔNp63 and miR-138-5p, which regulates SOX2, KLF4, and CD44 to promote the growth, metastasis and stemness of OSCC cells.

The dysregulation of TP63 has been reported in various human malignancies and its overexpression is closely associated with clinical outcomes [18, 19, 37, 38]. Generally, OSCC patients with elevated TP63 levels have a poorer survival rate. However, inconsistent results regarding the association of TP63 expression with OSCC patient prognosis have also been described [39]. These conflicting results may be attributed to the different demographic characteristics, anatomic sites [40], and spatiotemporal expression patterns of the TP63 isoforms in patients with OSCC. Our data suggest that TP63 (predominantly ΔNp63) likely plays a critical role in oral tumorigenesis and tumor progression. Intriguingly, the expression levels of TP63 and ΔNp63 were significantly higher in the tumor buddings (and the ITFs) than in their superficial or central counterparts. Tumor budding, which occurs in the stroma ahead of the ITFs, is defined as the presence of isolated single cells or small clusters of tumor cells [41]. Accumulating evidence suggests that the budding cells that detached from the tumor bulk represent a more aggressive sub-population of tumor cells with specific molecular characteristics[25, 42, 43]. In this study, increased TP63 expression was detected in the ITFs and tumor budding, which indicated that TP63 might play a pivotal role in tumor invasion and metastasis. Regardless, the practice of grouping OSCCs of various disease stages into a single group might conceal the complexity of a gene regulator (i.e., ΔNp63) that is involved in OSCC progression.

The stemness characteristics of HNSCC are linked to the aggressiveness of the tumor; these characteristics cause the tumor cells to acquire unlimited self-renewal potential, which promote subsequent tumor growth and metastasis [44]. Recent studies have demonstrated that TP63 regulates cancer stem cell properties by modulating Shh [15], NOTCH signaling [45], and miRNAs, including miR-34 [29], miR-205 [30], miR-200 [31], and miR-181 [32, 46]. Here, we show that ΔNp63 promotes a stem-like phenotype in OSCC cells by regulating SOX2, CD44, and KLF4 *in vitro* and *in vivo*. Furthermore, our data reveal that ΔNp63 transcriptionally suppresses miR-138-5p expression. Together, these findings suggest that ΔNp63 plays a crucial role in maintaining the self-renewal potential of tumor cells at least in part through miR-138-5p.

Our previous studies have shown that miR-138-5p inhibits migration and invasion capacity by governing the EMT in HNSCC cell lines [23, 47]. Additionally, miR-138-5p regulates SOX4, a determinant of the stemness phenotype, and inhibits invasion in ovarian cancer [48]. In agreement with these findings, our data show that miR-138-5p represses the stem-like properties of OSCC cells by regulating stemness-associated gene, such as SOX2, CD44, NOTCH1, and KLF4. Importantly, miR-138-5p does not regulate metastasis and stemness by itself; instead, it interacts with ΔNp63 to enable a crosstalk mechanism. These data are consistent with the mounting evidence shows that miRNAs form regulatory motifs with target genes to contribute to robustness during various biological processes [49]. Dysregulation of miRNAs in the regulatory network interferes with the maintenance of homeostasis and promotes tumor progression through increased proliferation, invasion and metastasis [50–54]. For example, in hepatocellular carcinoma, downregulation of miR-422a results in the increased expression of FOXG1/Q1/E1, target genes that transcriptionally inhibite miR-422a expression. The miR-422a-FOXG1/Q1/E1 feedback loop playes critical roles in hepatocellular carcinoma through its effects on cell proliferation and metastasis [52]. Zhang J et al. [53] found that PAX4 promotes migration and invasion in human epithelial cancers by transcriptionally inhibiting miR-144 and miR-451, which target the ADAM protein family members ADAMTS5 and ADAM10. This finding suggests that a PAX4-miR-144/451-ADAMs axis plays an important role in human epithelial cancer metastasis. In our study, it is likely that ΔNp63 transcriptionally suppresses miR-138-5p, and that miR-138-5p inhibits ΔNp63 expression in turn, forming a regulatory network. This crosstalk would significantly enhance the growth, invasion and stem-like properties of OSCC cells, which would explain the evident pro-metastasis phenotype in our study (Figure 7E).

We further validated the role of the interaction between ΔNp63 and miR-138-5p in OSCC tissues. As expected, a negative correlation between ΔNp63 and miR-138-5p was observed. Additionally, the OSCC patients with increased ΔNp63 expression but reduced miR-138-5p expression had the poorest prognoses. These findings imply that the interplay between ΔNp63 and miR-138-5p promotes tumor progression. However, it is important to note that oral tumorigenesis and metastasis are complicated processes that involve various regulators; one miRNA can affect the expression levels of thousands of genes. Hence, we cannot exclude the possibility that miR-138-5p regulates tumor progression through targets other than ΔNp63. As a transcription factor, TP63 (and ΔNp63) regulate the expression of hundreds of genes [32], which indicates that other genes and miRNAs may be involved in the interplay between ΔNp63 and miR-138-5p. Taken together, our data suggest that the capacity of ΔNp63 to promote tumor progression does not entirely depend on its interaction with miR-138-5p in OSCC cells. All these may be one explanation for the heterogeneity of OSCCs.

The major causes of tumor related deaths are local relapse and metastasis. Improvement in prognosis requires a better understanding of molecular pathogenesis of tumor patient so that targeted therapeutic interventions can be developed. In recent years, thanks to the development of precision medicine, targeting aberrant molecular pathways helps to personalize prognostication and treatment strategies. For example, hyperactive mTOR impairs cell differentiation via enhanced STAT3/p63/NOTCH signaling. Tumorigenic potential of cells with activated mTOR signaling are suppressed by NOTCH inhibition, which indicate that the STAT3/p63/NOTCH axis may serve as a target for the treatment of tumor with hyperactive mTOR signaling [55]. Additionally, Wang et al. [56] found that mTOR signaling activated in HNSCC with PIK3CA and RAS mutations causes resistance to cetuximab. Cotargeting mTOR with cetuximab may provide a promising therapeutic option for HNSCC patients. In our study, ΔNp63 promotes the OSCC progression cooperating with miR-138-5p, which may provide as a potential therapeutic target for OSCC patients.

## MATERIALS AND METHODS

### Patients and tissue specimens

Two OSCC patient cohorts were enrolled in this study. Cohort #1 samples (n=103) were obtained from the First Affiliated Hospital, Sun Yat-Sen University between June 2005 and June 2012. All OSCC specimens and 28 noncancerous adjacent tissues (NATs) were used for TP63 immunohistochemical assessment. Cohort #2 samples (n=110) were collected between June 2005 and August 2013 at the Hospital of Stomatology, Sun Yat-sen University. Immunohistochemical assays for ΔNp63 and *in situ* hybridization assays for miR-138-5p were performed on all 110 samples. All patients received radical surgery; none received any form of pre-surgical adjuvant therapy. Clinicopathological staging of the tumor was determined per the TNM classification system of UICC. DFS time was calculated from the date of surgery to the date of the final follow-up or cancer recurrence. The date of death was obtained from the medical records or follow-up telephone calls. All patients were given informed consent for aims of the research. The study was approved by the ethical committees of the Hospital of Stomatology and Sun Yat-Sen University.

### Immunohistochemical assay

Paraffin-embedded tumor tissues were cut into 4μm sections and processed for immunostaining. The sections were incubated overnight at 4°C with primary antibodies against TP63 (1:2000, EPR5701, Abcam), ΔNp63 (1:1000, N-16, Santa Cruz), Ki67 (1:1000, SP6, Abcam), SOX2 (1:200, D6D9, Cell Signaling Technology), CD44 (1:200, EPR1013Y, Abcam), KLF4 (1:200, D1F2, Cell Signaling Technology), and pan Cytokeratin (1:50, Novusbio), visualized using 3, 3-diaminobenzidine (DAB, Sigma-Aldrich) and counterstained with hematoxylin. Two senior pathologists who were blinded to the clinical data assessed and scored the IHC results. The fields were randomly selected for each sample under a light microscope with a 400× magnification. The staining index (SI) for TP63 (ΔNp63) was scored according to the staining intensity (0, no staining; 1, weak, light yellow; 2, moderate, yellow brown; 3, strong, brown) and the proportion of positive cells (0, 0%; 1, <10%; 2, <50%; 3, <75%; 4, ≥75%) per the following formula: SI= the proportion of positively stained cells×the staining intensity [57]. Cases with SI (TP63) >6 were classified into the high-expression group, and those with SI ≤6 were classified into the low-expression group. This scoring method was also used to evaluate the Ki67, SOX2, KLF4, and CD44 proteins expression in tumor xenografts.

### *In situ* hybridization

miR-138-5p expression was examined by *in situ* hybridization per the manufacturer's protocol (microRNA ISH Optimization Kit for FFPE, Exiqon, Vedbaek, Denmark). Briefly, after demasking, miR-138-5p was hybridized with Double-DIG-labeled LNA™ microRNA probes (1:000, Exiqon) overnight. The sections were incubated with an anti-digoxin monoclonal antibody (Roche Applied Science). The samples were counterstained with nitro blue tetrazolium/5-bromo-4-chloro-3-indolylphosphate (NBT/BCIP). The staining scores were determined based on both the intensity and proportion of the miR-138-5p-positive cells (in blue) in ten randomly selected fields for each specimen under a light microscope with a 400× magnification. The proportion of positively stained cells was graded as described above for TP63. The staining intensity of the cells was recorded as follows: 0 (no staining), 1 (weak, light blue), 2 (moderate, blue) and 3 (strong, dark blue). The staining index (SI) was calculated as SI=staining intensity×proportion of positively stained cells [58]. SI (miR-138-5p)>6 was defined as high expression, and SI≤6 was defined as low expression.

### Cell lines and cell culture

Human OSCC cell lines SCC9, SCC15, and CAL27 were obtained from ATCC (Rockville, MD, USA). UM1 was provided by Dr. Xiaofeng Zhou (University of Illinois at Chicago, IL, USA). HSC3, HSC6 and CAL33 and normal oral keratinocytes (NOK) were kindly provided by J. Silvio Gutkind (NIH, Bethesda, MD, USA). The HSC3, HSC6, CAL27, and CAL33 cells were cultivated in Dulbecco's modified Eagle's medium (DMEM, Gibco, Rockville, MD, USA) supplemented with 10% fetal bovine serum (FBS, Invitrogen, Carlsbad, CA, USA). The SCC9, SCC15, and UM1 cells were maintained in DMEM-F12 (Gibco) supplemented with 10% FBS. NOK was grown in keratinocyte serum-free medium containing human recombinant epidermal growth factor and bovine pituitary extract (Life Technologies). All cells were incubated at 37°C in a humidified atmosphere containing 5% CO2.

### Transient transfection

The TP63 or ΔNp63 siRNA (50nM, RiboBio, Guangzhou, China), miR-138-5p mimics (50nM, GE Dharmacon), miR-138-5p inhibitor (100nM, RiboBio), and their negative controls were transfected into OSCC cells using the Lipofectamine RNA iMAX Transfection Reagent (Invitrogen, CA, USA) according to the manufacturer's instructions. The TP63 siRNA sequences are shown in Supplementary Table 2.

### Viral vector constructs and stable cell lines establishment

The TAp63 lentiviral vector (pEZ-Lv201-TAp63), ΔNp63 lentiviral vector (pEZ-Lv201-ΔNp63) and control vector (pEZ-Lv201) were constructed by GeneCopoeia. The pEZ-Lv201-TAp63 plasmid encoded full-length human TAp63 complementary DNA (NM_003722.4), and the pEZ-Lv201-ΔNp63 plasmid encoded full-length human ΔNp63 complementary DNA (NM_001114980.1); both were sequence-verified. The ΔNp63 shRNA retroviral vector (pSR-ΔNp63 shRNA) and the negative control plasmid (pSR-shNC) were ordered from Addgene (MA, USA). To establish stable cell lines, the lentiviral vectors were packaged into pseudoviral particles and were delivered into SCC9 cells; cells were selected with 0.5μg/mL of puromycin (Sigma, St Louis, MO, USA) for four weeks. Similarly, recombinant retrovirus was generated by co-transfecting the ΔNp63 shRNA retroviral vector and the pIK plasmid into 293T cells, and used to infect SCC15 cells. The infected SCC15 cells were selected with 1μg/mL of geneticin (G418, Sigma, St Louis, MO, USA) for four weeks. The aforementioned shRNAs sequences are shown in Supplementary Table 2.

### Quantitative real-time PCR (qRT-PCR)

Total RNA was isolated from cells using the mirVana™ miRNA isolation kit (Ambion, USA). To determine the relative mRNA levels of TP63, miR-138-1, miR-138-2 and GAPDH, reverse transcription was performed using the Transcriptor First Strand cDNA Synthesis Kit (Roche). The PCR primer was purchased from Invitrogen (USA) and is listed in Supplementary Table 3. To detect the miRNAs levels, Bulgeloop™ miRNA qRT-PCR Primer Sets (including RT and qPCR primers) were synthesized by RiboBio. The qPCR reactions were performed using the SYBR GREEN I Master Mix or Light Cycler^®^480 Probes Master (Roche) on a Light Cycler 480 system (Roche) according to the manufacturer's instructions. The relative expression levels were calculated using the 2^− ΔΔCt^ method after normalization to the GAPDH or U6 expression levels.

### Western blot

Western blots were performed as previously described [23] using specific antibodies against TP63 (EPR5701, Abcam), SOX2 (D6D9), SOX4 (Millpore), NOTCH1 (D1E11), NOTCH3 (D11B8), NANOG (D73G4), OCT4, KLF4 (D1F2), CD44 (EPR1013Y, Abcam), and ABCG2; GAPDH (1:2000, 14C10) and α-Tubulin (1:2000, 11H10) were used as internal references. All antibodies were purchased from Cell Signaling Technology, except where indicated, and used at 1:1000 dilutions. The full scans of the Western blots were quantified using the Alpha Innotech imaging software (San Leandro, CA, USA).

### Migration and invasion assays

The migration assay and invasion assays were performed as previously described [25]. Briefly, for migration assays, 4×10^4^ cells were seeded into the upper chambers containing serum-free medium and non-coated membranes (24-well insert; pore size, 8μm; BD Biosciences); 8×10^4^ cells were seeded into the upper chambers with Matrigel-coated membranes for the invasion assays. The lower chambers were filled with medium containing 10% FBS. The cells were incubated for 24h at 37°C. The cells that had not traversed the membrane were gently removed using a cotton swab, and the cells on the lower surface of the membrane were stained with 0.1% crystal violet (Sigma) and observed under a Zeiss microscope.

### Cell proliferation assay

Cell proliferation was analyzed using the Cell Counting Kit-8 (CCK-8, Sigma-Aldrich, Santa Clara, CA, USA). Briefly, 2×10^3^cells were seeded in triplicate into a 96-well plate. Cell viability was assessed at 1, 2, 3, 4, 5 and 6 days post-transfection. The absorbance was measured at 450nm using a microplate reader (Genios TECAN, Männedorf, Schweiz).

### Colony formation assays

For colony formation assays, 5×10^2^ cells were seeded into 6-well plates at 24h post-transfection. After culturing for 10 days, visible colonies were stained with crystal violet. Colonies with diameters above 1 mm were counted.

### Tumor sphere-forming assay

One thousand cells (SCC9 or SCC15) were seeded into ultra-low attachment 6-well plates in serum-free DMEM/F12 medium supplemented with 2% B27 (Invitrogen), 10 ng/mL epidermal growth factor (EGF, Invitrogen), 10 ng/mL basic fibroblast growth factor (bFGF, Invitrogen), and 1% penicillin/streptomycin. The medium was changed every three days until tumor sphere formation was observed (approximately 2 weeks). The sphere diameters were assessed by a stereomicroscopy (ZEISS).

### ALDH activity and CD133 staining

The ALDH activity was measured by using ALDEFLUOR™ detection kit (StemCell Technology, 01700) according to the manufacturer's instructions and data were acquired on CytoFLEX (Beckman Coulter). CD133 staining was carried out using CD133 (AC133) antibody (MACS Miltenyi Biotech). The cells were then washed twice with staining buffer and measured on CytoFLEX. Analyses were performed using CytExpert software (Beckman Coulter).

### *In vivo* experiments

All animal experiments were approved by the ethical committees of Sun Yat-Sen University and followed the protocol. Four- to six-week-old female BALB/c nude mice were purchased from the Animal Care Unit of Guangdong, China and maintained in pathogen-free conditions.

For subcutaneous injections, 5×10^6^ cancer cells were injected into the flank region of each nude mouse, with 5 mice per group. Tumor growth was routinely observed. Tumor volume (mm^3^) was measured every 7 days using vernier calipers and calculated using the following formula: V = L×W^2^/2, where L represents length and W represents width. The mice were sacrificed, and the tumors were collected and weighed at the end of 7 weeks after injection.

For intravenous injections, 2×10^6^ SCC9 cells were injected into each nude mouse, with 5 mice per group. The mice were sacrificed and liver metastases were evaluated when one of them became moribund.

### Chromatin immunoprecipitation assay (ChIP)

ChIP was performed using a ChIP assay kit (Millipore) according to the manufacturer's instructions. Briefly, cancer cells were incubated with 1% formaldehyde for 10 min at room temperature to crosslink their DNA. The cells were lysed in lysis buffer and sonicated to generate DNA fragments between 500 bp and 800 bp in length as assessed by agarose gel electrophoresis. Cross-linked chromatin was incubated at 4°C overnight with an antibody against TP63 (D2K8X, Cell Signaling Technology) or the isotype control (IgG). Before the IP, a sample of the input DNA was collected for normalization. After reversing the DNA–protein crosslinks, the immunoprecipitated DNA was purified using the QIAquick PCR purification kit (Qiagen) following the manufacturer's protocol. The final precipitated DNA was subjected to qPCR reactions using specific primers for the TP63 binding sites in the human miR-138-2 or TP21 (positive control [59]) promoters. The primers are listed in Supplementary Table 3. The PCR result was normalized using the input DNA, and the specificity of the PCR amplification was confirmed by agarose gel electrophoresis.

### Dual luciferase reporter assay

To validate the TP63 binding sites in the miR-138-2 promoter, the miR-138-2 promoter reporter constructs with the wild-type (pGL3-Wt) or mutated (pGL3-Mut-BS1, pGL3-Mut-BS2, pGL3-Mut-BS1+2) TP63 binding sites were co-transfected with the pRL-SV40 Renilla luciferase vectors into OSCC cells using the Lipofectamine^®^ 3000 Transfection Reagent (Invitrogen). To validate whether TP63 was a direct target of miR-138-5p, TP63 luciferase reporter pmiR-GLO (Vec) constructed with the wild-type (Wt1 and Wt2) or mutated miR-138-5p binding sites (Mut1 and Mut2) were transfected into SCC15 cells. Luciferase activity assays were performed 48 hours after the transfections using the Dual-Luciferase Reporter Assay System (Promega) on a Lumat LB 9507 Luminometer (Promega) per the manufacturer's recommendation. Measurements from triplicate transfections were analyzed after normalization to the Renilla luciferase activities as previously described [23].

### MicroRNA microarray analysis

To determine the different microRNA expression profiles between SCC9 Lv-NC and Lv-ΔNp63 cells, Exiqon miRCURY-Ready-to-Use PCR-Human-panel-I+II-V4.M microarrays (Exiqon, Vedbaek, Denmark), which covered 752 high-priority or most important human microRNAs, were used per the manufacturer's instructions. Briefly, 25 ng of RNA was extracted from the sample, reverse transcribed using the Universal cDNA Synthesis Kit II (Exiqon) and subjected to an Exiqon miRCURY-Ready-to-Use PCR panel (Exiqon) with ExiLENT SYBR® Green master mix (Exiqon) on a 7900HT real-time PCR system (Applied Biosystems, Foster City, CA, USA). Each assay measurement that was accompanied by appropriate melting curves and a Ct<35 was included in the data analysis. Normalization was performed using three small RNA reference genes, including U6, SNORD38B and SNORD49A, per the following formula: Ct (Δ Ct) = average Ct (assay) – average Ct (normalizer assays). The relative expression between the SCC9 Lv-ΔNp63 and Lv-NC cells was assessed using the 2^−ΔΔCt^ method.

### Bioinformatics analysis

Gene expression was analyzed using microarray gene expression datasets from the Oncomine data-base (https://www.oncomine.org). The significantly dysregulated TP63 gene in the OSCCs was identified based on the following 4 microarrays: the Estilo Head-Neck microarray (31 tongue squamous cell carcinomas, TSCCs vs. 26 tongue tissues); the Talbot Lung microarray (31 TSCCs vs. 26 tongue tissues); the Ye Head-Neck microarray (26 TSCCs vs. 12 tongue tissues) and the Schlingemann Head-Neck microarray (4 HNSCCs vs. 4 Oropharynx or hypopharynx tissues). We also analyzed TP63 gene expression in the following datasets from other cancer types: the Hou Lung microarray (27 lung cancers vs. 65 lung tissues); the Wachi Lung microarray (5 lung cancers vs. 5 lung tissues); the Garber Lung microarray (13 lung cancers vs. 5 lung tissues); the Bhattacharjee Lung microarray (21 lung cancers vs. 17 lung tissues); the Su Esophagus microarray (53 esophagus squamous cell carcinomas vs. 53 esophagus tissues); the Finak Breast microarray (53 breast cancers vs. 6 breast tissues); the Sanchez-Carbayo Bladder microarray (28 superficial bladder cancers vs. 48 bladder tissues) and the Nindl Skin microarray (5 skin squamous cell carcinomas vs. 6 skin tissues). The gene expression data in the datasets from each array were log_2_ transformed and median centered; the standard deviation (SD) was normalized to one for each array. The TP63 gene expression values in each dataset were read from the displayed bar chart, recorded into Excel and analyzed.

### Statistical analysis

All statistical analyses were performed using SPSS 20.0 (SPSS Inc., Chicago, IL, USA) or the GraphPad Prism 6.0 software (La Jolla, CA, USA). Two-group comparisons were analyzed using Student's t-test, the Wilcoxon test or the χ2 test; multiple-group comparisons were assessed by one-way analysis of variance (ANOVA). Pearson correlation analyses were used to determine the correlation between TP63 and miR-138-5p expression. The Kaplan–Meier method and log-rank test were performed to determine the survival outcomes. Univariate and multivariate analyses were performed using a Cox proportional regression hazards model. Hazard ratios and 95% confidence intervals were derived from the model, and the likelihood ratio test was used to compare the groups. Differences were considered significant at *P* < 0.05.

## SUPPLEMENTARY MATERIALS FIGURES AND TABLES



## Abbreviations

OSCC: oral squamous cell carcinoma
TSCC: tongue squamous cell carcinoma
HNC: head and neck cancer
miRNA: microRNA
miR-138-5p: microRNA-138-5p
3′UTR: 3′ terminal untranslated regions
NAT: noncancerous adjacent tissues
qRT-PCR: real-time quantitative reverse-transcription polymerase chain reaction
H&E: hematoxylin and eosin.
