# Supplementary material for: Interplay between ΔNp63 and miR-138-5p regulates growth, metastasis and stemness of oral squamous cell carcinoma
Source: Oncotarget. 2017 Feb 27;8(13):21954–73. doi: 10.18632/oncotarget.15752 (PMC5400637; doi:10.18632/oncotarget.15752)
Supplement: Supplementary file 1 [file oncotarget-08-21954-s001.pdf]

Interplay between ΔNp63 and miR-138-5p regulates growth, metastasis and stemness of oral squamous cell carcinoma

SUPPLEMENTARY MATERIALS

SUPPLEMENTARY FIGURES AND TABLES

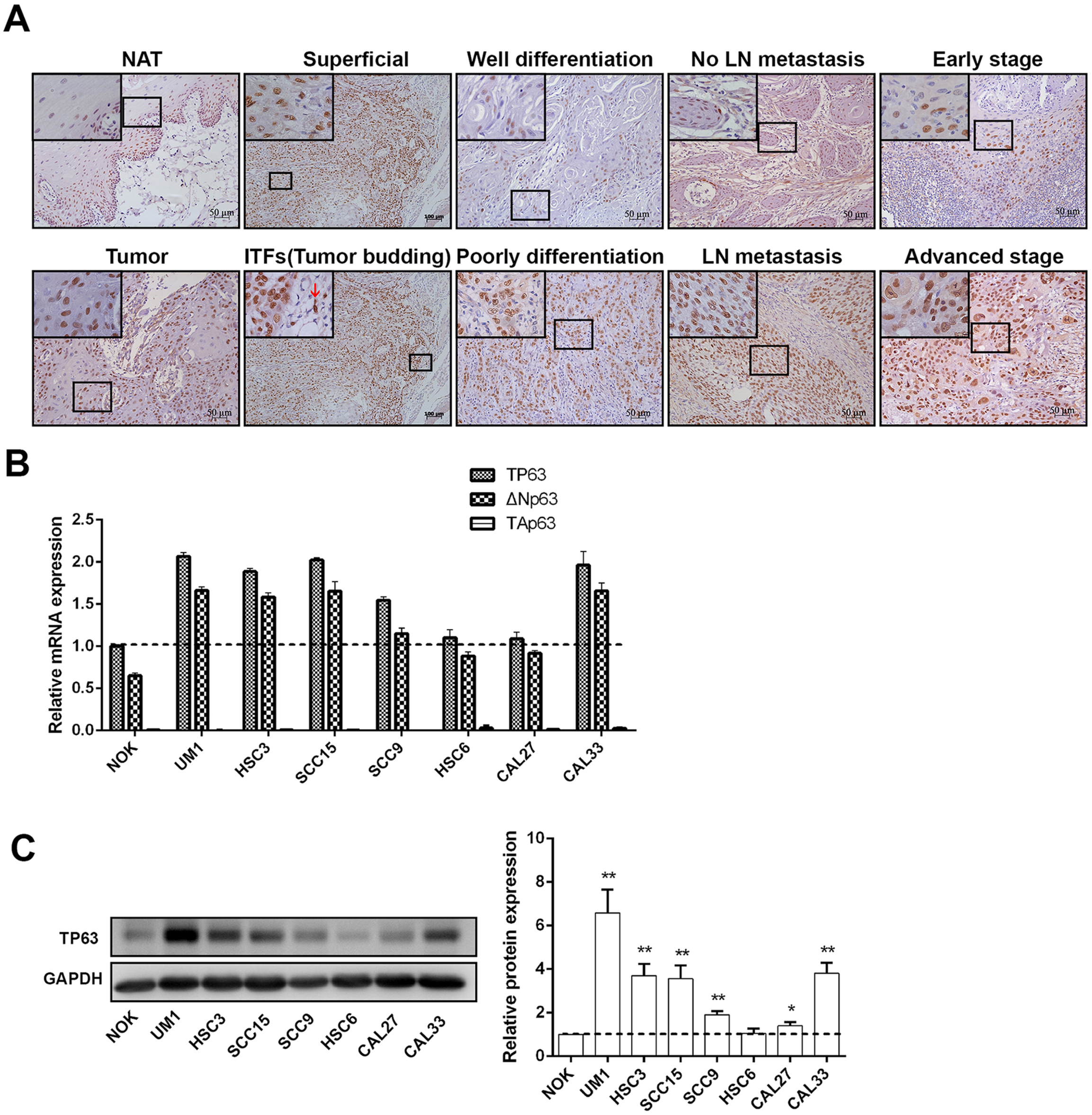

Supplementary Figure 1: TP63 is up-regulated in OSCC samples and cell lines. A. ΔNp63 expression in OSCC tissues and noncancerous adjacent tissues. Representative immunohistochemistry images for ΔNp63 staining in NATs, different localizations in one OSCC tissue and OSCC tissues from various pathological differentiation, lymph nodes statuses and clinical stages are shown. Red arrows represent tumor budding cells. Original magnification 400×. **B.** The mRNA expression levels of TP63, TAp63, and ΔNp63 in normal oral keratinocyte (NOK) and OSCC cell lines were analyzed by qRT-PCR. **C.** The TP63 protein expression levels in NOK cells and OSCC cell lines were determined by Western blot (left panel). Quantification of TP63 protein expression levels (right panel). The results were presented as the mean±SD obtained from three independent experiments. **P*<0.05, ***P*<0.01.

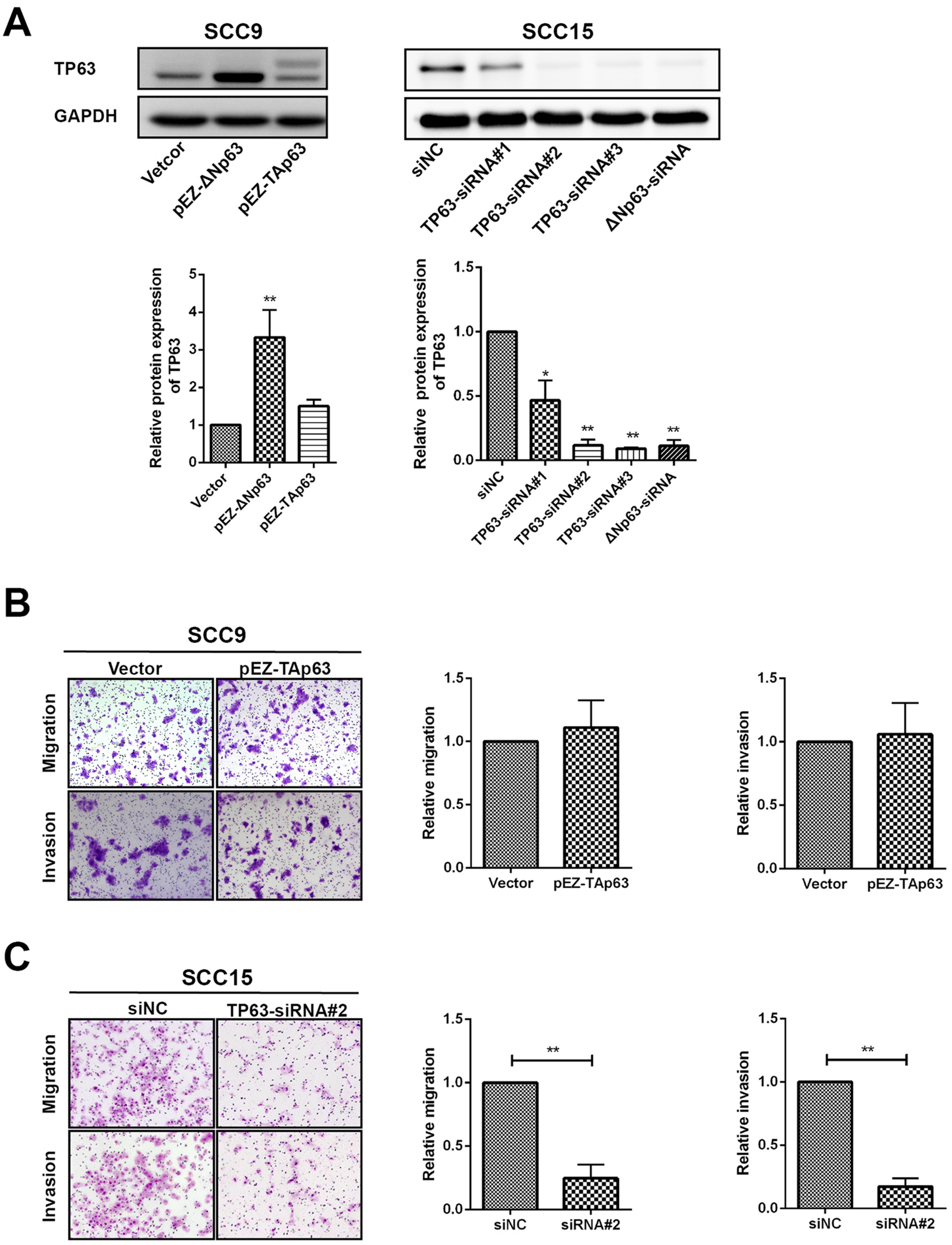

Supplementary Figure 2: TP63 promotes OSCC metastasis *in vitro*. A. The transfection efficiency in TP63 overexpression and knockdown in indicated cells was determined by Western blot. **B.** Transwell assays showing the capacity of migration or invasion in SCC9 cells after enhancing TAp63 expression (left panel). Quantification for the relative number of migrated or invaded cells (right panel). **C.** Transwell assays showing the capacity of migration or invasion in SCC15 cells after TP63 knockdown (left panel). Quantification for the relative number of migrated or invaded cells (right panel). The results were presented as the mean±SD obtained from three independent experiments. *P<0.05, **P<0.01.

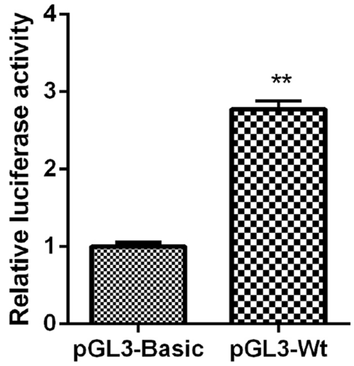

Supplementary Figure 3: Validation of the activity of miR-138-2 promoter. Transfection of a reporter plasmid that contained the miR-138-2 promoter (pGL3-Wt) showed an increase in luciferase activity compared with the control reporter (pGL3-Basic) in 293T cells. The results were presented as the mean±SD of three independent experiments. ***P*<0.01.

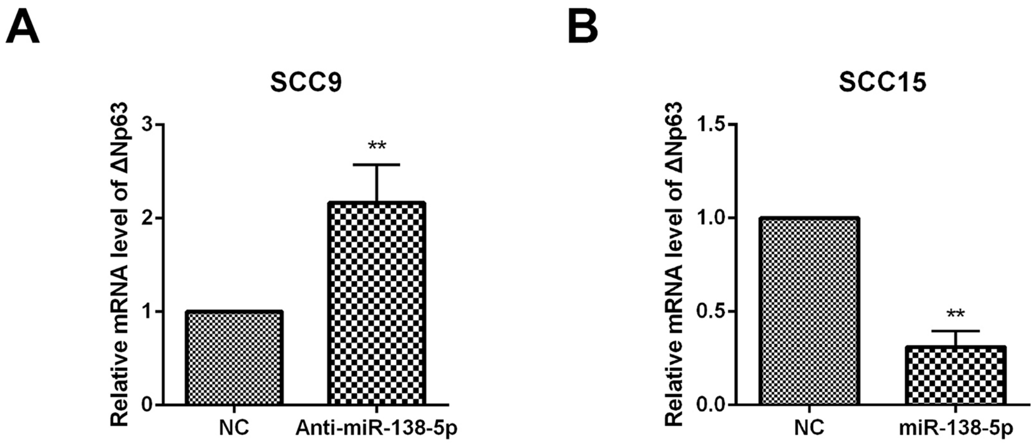

Supplementary Figure 4: ΔNp63 is a direct target of miR-138-5p. A. The qRT-PCR showing the ΔNp63 mRNA expression levels in SCC9 cells transfected with miR-138-5p Inhibitors (Anti-miR-138-5p) or control (NC). **B.** The qRT-PCR showing the ΔNp63 mRNA expression levels in SCC15 cells transfected with miR-138-5p mimics or control (NC). The results were presented as the mean±SD obtained from three independent experiments. ***P*<0.01.

Supplementary Table 1: Clinicopathological features of OSCC patients in cohort #2

Characteristic	Subcharacteristic	n	%
**Gender**	Female	51	46.4
	Male	59	53.6
**Age**	≤55	59	53.6
	>55	51	46.4
**Tumor size**	≤4cm	79	71.8
	>4cm	31	28.2
**T classification**	T_1-T_2	61	55.5
	T_3-T_4	49	44.5
**Pathological differentiation**	Well	66	60.0
	Moderately/Poorly	44	40.0
**LN metastasis**	Negative	69	62.7
	Positive	41	37.3
**Clinical stage**	I-II	44	40.0
	III-IV	66	60.0
**ΔNp63 expression**	Low	50	45.5
	High	60	54.5
**MiR-138-5p expression**	Low	57	51.8
	High	53	48.2

Abbreviations: LN, Lymph node.

Supplementary Table 2: Sequences of siRNA and shRNA

Gene		Sequences (5′-3′)
**TP63 siRNA#1**	**Sense**	GGAACUCAUGCAGUACCUU dTdT
	**Antisense**	dTdT CCUUGAGUACGUCAUGGAA
**TP63 siRNA#2**	**Sense**	CCACUGAACUGAAGAAACU dTdT
	**Antisense**	dTdT GGUGACUUGACUUCUUUGA
**TP63 siRNA#3**	**Sense**	CGUCAGAACACACAUGGUA dTdT
	**Antisense**	dTdT GCAGUCUUGUGUGUACCAU
**ΔNp63 siRNA**	**Sense**	AGGACAGCAGCAUUGAUCA dTdT
	**Antisense**	dTdT UCCUGUCGUCGUAACUAGU
**ΔNp63 shRNA**	**Sense**	CCCCTGCCAAATTGCAAAGACATGTTCA AGAGACATGTCTTTGCAATTTGGCAGTTTTT
	**Antisense**	AAAAACTGCCAAATTGCAAAGACATG TCTCTTGAACATGTCTTTGCAATTTGGCAGGGG

Supplementary Table 3: Sequences or ID of primers

**Primers used for qRT-PCR**	
**Gene**	**Sequences (5′-3′) or assay ID**
TP63-Forward	TAACACAGACCACGCGCAGA
TP63-Reverse	GAATACGTCCAGGTGGCCGA
TAp63- Forward	GGACTGTATCCGCATGCAG
TAp63- Reverse	GAGCTGGGCTGTGCGTAG
ΔNp63-Forward	GAAGAAAGGACAGCAGCATTGA
ΔNp63- Reverse	GGGACTGGTGGACGAGGAG
GAPDH-Forward	GGACCTGACCTGCCGTCTAG
GAPDH-Reverse	GTAGCCCAGGATGCCCTTGA
miR-138-1	Hs03303255_pri
miR-138-2	Hs03303145_pri
GAPDH	Hs03929097_g1
**Primers used for CHIP-PCR**	
Bind site 1-Forward	GGGTTCGAGTCCTTGGTTT3
Bind site 1-Reverse	ATCCCCATCAACTCTTTATCTG
Bind site 2-Forward	TCTTCCCACTTCAGCCTCCC
Bind site 2-Reverse	TGGTGCCTCACGCCTGTAAT
TP21-Forward	AGCAGGCTGTGGCTCTGATT
TP21-Reverse	CAAAATAGCCACCAGCCTCTTCT
